# Surgeons’ Experience and Practices in Managing the Pilonidal Sinus in the Kurdistan Region of Iraq

**DOI:** 10.7759/cureus.62115

**Published:** 2024-06-10

**Authors:** Rawand M Haweizy

**Affiliations:** 1 Department of Surgery, College of Medicine, Hawler Medical University, Erbil, IRQ

**Keywords:** treatment, method, recurrence, experience, surgeon, pilonidal sinus

## Abstract

Background

Despite pilonidal sinus disease being a prevalent issue, there are still many challenges and controversies regarding its management. This study aimed to evaluate the experiences and practices of surgeons in the Kurdistan region of Iraq in the management of the pilonidal sinus and determine the most preferred treatment method, recurrence rates, and other complications related to different treatment methods.

Methods

This cross-sectional study was conducted on a convenience sample of 104 surgeons in the Kurdistan region of Iraq from January to February 2024 using an online survey based on Google Forms. A questionnaire was developed for data collection that included data on the experience and practice of pilonidal sinus treatment.

Results

The most common procedure followed by the study participants to manage the pilonidal sinus included primary open (n = 61/104, 58.7%), followed by primary closure (n = 20/104, 19.2%). The most common reasons or advantages for performing specific procedures to manage the pilonidal sinus were a lower recurrence rate (n = 73/104, 70.2%), safer procedures (n = 60/104, 57.7%), shorter operation times (n = 57/104, 54.8%), and shorter hospital stays (n = 53/104, 51.0%). The primary open method was the most commonly used method (n = 46/104, 44.3%), followed by simple incision and drainage (n = 25/104, 24.0%), primary closure (n = 23/104, 22.1%), and off-midline closure (n = 10/104, 9.6%). Most of the participants agreed that the primary open had the lowest recurrence rate (n = 68/104, 65.4%), while simple incision and drainage (n = 50/104, 48.1%) and primary closure (n = 29/104, 27.9%) were associated with frequent recurrence.

Conclusions

Standard treatment of pilonidal sinus disease is still not available. Most surgeons in the Kurdistan region of Iraq prefer the open method, which is the easiest, safest, and least recurrent yield method. However, it is the most painful and has the longest recovery time.

## Introduction

Pilonidal sinus disease is an acute or chronic infection in the subcutaneous fatty tissue in the natal cleft. The incidence of infected pilonidal sinus disease is approximately 25/100,000, affecting nearly 0.7% of the population. The disease is more common in men than in women and is most frequently affecting those aged 15-30. It is rare before puberty or after 60 [1].

Common predisposing factors to pilonidal sinus disease include overweight, hairy body, thick skin, a deep gluteal cleft, sitting position for several hours a day, lack of hygiene, and family history [2,3]. Pilonidal sinus disease is one of the most common surgical problems among young adults. Excess sweating and excessive body hair are the main risk factors for pilonidal sinus disease [4,5]. Pilonidal sinus disease is common in young adults, especially men with intergluteal cleft or deep natal and coarse body hair [6].

Pilonidal sinus disease has increased in incidence in the last 50 years for unknown reasons, especially in young European and North American men [7]. An accepted milestone is the period 1833-1880 during which awareness of the disease was gained. In 1880, it was given its name, although "Jeep Disease" is a common way to refer to it [8]. Due to the various ways in which the disease presents clinically, it is necessary to tailor treatments according to the specific patient and the severity of the disease they carry [9].

Pilonidal sinus disease is clinically asymptomatic in some cases but can also present as a chronic and complicated disease with multiple sinus tracts that could severely affect the patient's quality of life. The heterogeneous nature of the presentation of pilonidal sinus disease is related to the progressive course of development of the pilonidal sinus [10].

Surgical methods for treating pilonidal sinus disease range from wide excision, with or without primary closure, to various flap closures. These surgical techniques aim to eliminate the underlying causes driven by natal cleft hair and reduce recurrence. However, the five-year long-term recurrence rate is 10-30%, with significant complication rates. There are multiple techniques to treat pilonidal sinus disease, but there is no universally accepted technique. The rate of recurrence of pilonidal sinus disease is high even after treatment [5].

An ideal treatment for pilonidal sinus disease has not yet been discovered. The most commonly practiced technique includes simple closure following a resection of the affected tissue. However, the high recurrence rates required the search for other methods, including the V-Y advancement flap (VYAF), which in theory results in the flattening of the natal cleft without tension in the suture line [11]. The best surgical technique for pilonidal sinus disease remains controversial [12].

Despite pilonidal sinus disease being a prevalent issue, there are still many challenges and controversies regarding its management. Common surgery methods that many surgeons perform to target this disease are excisions and laying open to heal with secondary intention since many surgeons believe that such procedures are associated with fewer recurrence rates and complications [13]. Currently, advances in treatment are being introduced, such as flaps and laser ablation, but these are not yet recognized methods [14].

There is limited research from Iraq and the Kurdistan region of Iraq regarding the preferred methods of managing pilonidal sinus disease among surgeons. Available research shows that the open method and closed method are equally practiced (56% and 44%, respectively). The closed method is preferred for being better in terms of recovery, hospital stay, and time off work, while the open method is superior for a lower recurrence rate [15]. This study aimed to assess the experience and practices of surgeons in the Kurdistan region of Iraq in managing the pilonidal sinus and to determine the most preferred treatment method. It also aimed to assess the surgeon's experience with recurrence rates and other complications related to different treatment methods.

## Materials and methods

Design and setting

This cross-sectional study was carried out in the Kurdistan region of Iraq from January to February 2024 using an online survey based on Google Forms.

Participants

The sample size was calculated based on a population of 230 surgeons in the Kurdistan region of Iraq, the most extreme prevalence of 50% with a 95% confidence interval and ±5% precision. A sample size of 144 women was calculated, which was increased to 160 to account for nonresponse.

Due to a lack of complete lists of all surgeons in the Kurdistan region of Iraq with contact details, a random sample could not be selected. Therefore, a convenience sample of surgeons was selected and invited to participate in the study. The sample was selected from the lists of the local association of surgeons and the directorates of health and medical schools in the region. Consultants, specialists, and general surgeons of physicians were included in the study.

Study tool and data collection

A questionnaire was developed for data collection based on a literature review and experts' opinions. The questionnaire included two sections. The first section included questions about the demographic and professional information of the participants, such as age, sex, highest education level, specialty level, affiliation, place of work, and years of experience as a surgeon. The second section included data on the experience and practice of pilonidal sinus treatment, including the number of cases of pilonidal sinus treated by the surgeon, the procedure performed most frequently, reasons for the preferred procedure, how often each procedure is performed (never to very frequently), and the rate of recurrence and the rate of other complications of each type of procedures (very low to very high). The study questionnaire was tested on 10 participants to assess its clarity, comprehensibility, acceptance, and internal consistency.

The online survey tool was shared with the study sample through the Viber and WhatsApp groups of surgeons in the Kurdistan region of Iraq.

Ethical aspects

Participants were provided with a description of the study and informed about its voluntary participation and anonymity before requesting written online consent. The study protocol was approved by the Research Ethics Committee of the College of Medicine of Hawler Medical University.

Statistical analysis

Data were analyzed using Statistical Product and Service Solutions (SPSS, version 22; IBM SPSS Statistics for Windows, Armonk, NY). Frequencies and percentages were calculated and displayed in tables. Questionnaires or sections with missing data were excluded from the analysis.

## Results

A total of 104 surgeons responded to the survey and completed the online questionnaire (response rate 65%). The highest proportion of the participants were male (n = 90/104, 86.5%), board degree holders (n = 61/104, 58.57%), specialist surgeons (n = 62/104, 59.6%), affiliated with the directorate of health (n = 62/104, 59.6%), from the Erbil governorate (n = 66/104, 63.5%), and working within the main cities (n = 81/104, 77.9%). Regarding the number of pilonidal sinus cases managed, 43 (41.3%) participants had managed 101-500 cases, and 21 (20.2%) had managed 51-100 cases (Table 1).

**Table 1:** Demographic and professional characteristics of the surgeons who participated in the study (n=104)

Variable	No.	(%)
Sex		
Male	90	(86.5)
Female	14	(13.5)
Education		
MBChB	16	(15.4)
High diploma	23	(22.3)
Board	61	(58.7)
PhD	4	(3.8)
Specialty level		
Practitioner	16	(15.4)
Specialist	62	(59.6)
Consultant	26	(25.0)
Affiliation		
Directorate of health	62	(59.6)
University	35	(33.7)
Private sector	7	(6.7)
Governorate		
Erbil	66	(63.5)
Duhok	9	(8.7)
Suly	29	(27.9)
Place or work		
Inside main cities	81	(77.9)
Outside main cities	23	(22.1)
Number of PNS cases managed		
≤10	4	(3.8)
11-50	17	(16.3)
51-100	21	(20.2)
101-500	43	(41.3)
>500	19	(18.3)
Total	104	(100.0)

The most common procedure followed by the study participants to manage the pilonidal sinus included primary open (n = 61/104, 58.7%) and then primary closure (n = 20/104, 19.2%). The other procedures were not used commonly, as shown in Table 2.

**Table 2:** Procedures most performed by surgeons for the treatment of the pilonidal sinus

Procedure	No.	(%)
Primary open	61	(58.7)
Primary closure	20	(19.2)
Off-midline closure	4	(3.8)
Marsupialization	4	(3.8)
Simple incision and drainage	3	(2.9)
Karydakis flap	3	(2.9)
Limberg flap	3	(2.9)
Laser therapy	3	(2.9)
Partial closure	2	(1.9)
Excision secondary closure	1	(1.0)
Total	104	(100.0)

As shown in Table 3, the most common reasons or advantages for performing specific procedures to manage the pilonidal sinus were a lower recurrence rate (agree and strongly agree n = 73/104, 70.2%), safer procedures (n = 60/104, 57.7%), shorter operation times (n = 57/104, 54.8%), and shorter hospital stays (n = 53/104, 51.0%).

**Table 3:** Reasons or advantages for performing specific procedures for managing the pilonidal sinus

Reasons	Participants’ agreement (n, %)												
	Strongly Disagree		Disagree		Neutral		Agree		Strongly Agree		Not Applicable		
Less recurrence rate	11	(10.6)	2	(1.9)	17	(16.3)	45	(43.3)	28	(26.9)	1	(1.0)	
Safer	7	(6.7)	5	(4.8)	29	(27.9)	48	(46.2)	12	(11.5)	3	(2.9)	
Shorter operation time	9	(8.7)	10	(9.6)	27	(26)	40	(38.5)	17	(16.3)	1	(1.0)	
Shorter hospital stay	10	(9.6)	8	(7.7)	30	(28.8)	34	(32.7)	19	(18.3)	3	(2.9)	
Less wound dehiscence	11	(10.6)	16	(15.4)	27	(26.0)	36	(34.6)	11	(10.6)	3	(2.9)	
Less wound infection	12	(11.5)	15	(14.4)	33	(31.7)	29	(27.9)	14	(13.5)	1	(1.0)	
Shorter durations of return to work	17	(16.3)	29	(27.9)	25	(24.0)	20	(19.2)	10	(9.6)	3	(2.9)	
Less pain	15	(14.4)	30	(28.8)	37	(35.6)	16	(15.4)	5	(4.8)	1	(1.0)	

Regarding the frequent procedures used by surgeons, primary open was the most frequently used (frequently and very frequently n = 46/104, 44.3%), followed by simple incision and drainage (n = 25/104, 24.0%), primary closure (n = 23/104, 22.1%), and off-midline closure (n = 10/104, 9.6%). The other procedures were rarely or never used, as shown in Table 4.

**Table 4:** The frequency of using different procedures by surgeons to treat the pilonidal sinus

Procedure	Frequency of using different procedures (n, %)											
	Never		Rarely		Sometimes		Frequently		Very frequently		Always	
Primary open	8	(7.7)	12	(11.5)	26	(25.0)	32	(30.8)	14	(13.5)	12	(11.5)
Primary closure	16	(15.4)	26	(25.0)	35	(33.7)	13	(12.5)	10	(9.6)	4	(3.8)
Simple incision and drainage	7	(6.7)	20	(19.2)	52	(50.0)	22	(21.2)	3	(2.9)	0	(0.0)
Off-midline closure	45	(43.3)	24	(23.1)	23	(22.1)	9	(8.7)	1	(1.0)	2	(1.9)
Laser therapy	54	(51.9)	25	(24.0)	16	(15.4)	5	(4.8)	3	(2.9)	1	(1.0)
Limberg flap	65	(62.5)	25	(24.0)	9	(8.7)	1	(1.0)	3	(2.9)	1	(1.0)
Karydakis flap	70	(67.3)	20	(19.2)	9	(8.7)	2	(1.9)	2	(1.9)	1	(1.0)
Phenol injections	90	(86.5)	7	(6.7)	4	(3.8)	1	(1.0)	1	(1.0)	1	(1.0)
Modified Limberg flap	83	(79.8)	13	(12.5)	6	(5.8)	1	(1.0)	0	(0.0)	1	(1.0)

Most of the participants agreed that primary open had the lowest recurrence rate (very low and low n = 68/104, 65.4%), while simple incision and drainage (high and very high n = 50/104, 48.1%) and primary closure (n = 29/104, 27.9%) were associated with frequent recurrence. The participants were not sure of the recurrence rate of the other procedures. Regarding complications other than recurrence, participants thought primary open was associated with the least frequent complications (very low and low n = 56/104, 53.8%). Primary closure (high and very high n = 26/104, 25.0%) and simple incision and drainage (n = 21/104, 20.2%) were associated with more frequent complications. Participants were not sure of the frequency of other complications associated with the remaining procedures. Table 5 shows the participants' experience with the frequency of recurrence and other complications of the different procedures.

**Table 5:** The frequency of recurrence and other complications of different pilonidal sinus procedures from the experience of the participants

Procedure	Frequency of complications (n, %)											
	Very low		Low		Neutral		High		Very high		Not sure	
	Recurrence rate											
Primary open	37	(35.6)	31	(29.8)	21	(20.2)	5	(4.8)	3	(2.9)	7	(6.7)
Primary closure	3	(2.9)	26	(25)	40	(38.5)	28	(26.9)	1	(1.0)	6	(5.8)
Simple incision and drainage	2	(1.9)	15	(14.4)	24	(23.1)	41	(39.4)	9	(8.7)	13	(12.5)
Off-midline closure	5	(4.8)	25	(24.0)	26	(25.0)	8	(7.7)	3	(2.9)	37	(35.6)
Laser therapy	7	(6.7)	21	(20.2)	14	(13.5)	6	(5.8)	4	(3.8)	52	(50.0)
Limberg flap	9	(8.7)	25	(24.0)	18	(17.3)	0	(0.0)	1	(1.0)	51	(49.0)
Karydakis flap	8	(7.7)	19	(18.3)	16	(15.4)	1	(1.0)	2	(1.9)	58	(55.8)
Phenol injections	5	(4.8)	0	(0.0)	20	(19.2)	11	(10.6)	5	(4.8)	63	(60.6)
Modified Limberg flap	7	(6.7)	21	(20.2)	16	(15.4)	0	(0.0)	2	(1.9)	58	(55.8)
	Other complications											
Primary open	27	(26.0)	29	(27.9)	27	(26.0)	6	(5.8)	4	(3.8)	11	(10.6)
Primary closure	10	(9.6)	24	(23.1)	30	(28.8)	23	(22.1)	3	(2.9)	14	(13.5)
Simple incision and drainage	10	(9.6)	31	(29.8)	27	(26.0)	18	(17.3)	3	(2.9)	15	(14.4)
Off-midline closure	4	(3.8)	22	(21.2)	27	(26.0)	4	(3.8)	3	(2.9)	44	(42.3)
Laser therapy	14	(13.5)	13	(12.5)	22	(21.2)	2	(1.9)	5	(4.8)	48	(46.2)
Limberg flap	4	(3.8)	18	(17.3)	26	(25.0)	2	(1.9)	2	(1.9)	52	(50.0)
Karydakis flap	5	(4.8)	16	(15.4)	20	(19.2)	5	(4.8)	2	(1.9)	56	(53.8)
Phenol injections	4	(3.8)	12	(11.5)	19	(18.3)	4	(3.8)	2	(1.9)	63	(60.6)
Modified Limberg flap	4	(3.8)	16	(15.4)	22	(21.2)	2	(1.9)	3	(2.9)	57	(54.8)

## Discussion

Controversy still surrounds the question of what the best surgical technique for sacrococcygeal pilonidal disease is [12]. This study aimed to find the most preferred surgical method of treatment of pilonidal sinus disease among surgeons in the Kurdistan region of Iraq, in addition to recognizing which method is associated with lower recurrence rates and fewer complications.

Regarding the demographic patterns of the surgeons in the Kurdistan region of Iraq, the ratio of male-to-female surgeons is 90:14 (86.5, 13.5%). A study by Hesse in North America showed similar results, with only 20.3% of surgeons being female [16]. Surgery has traditionally been considered a highly male-dominated specialty despite the increasing number of women who have graduated from medical schools over the years. This theme appears similar in developed and developing countries [5]. Most surgeons (n = 62/104, 59.6%) have a specialty level, followed by a consultant level (n = 26/104, 25%). According to the Ministry of Health (MOH) laws and guidelines, in the Kurdistan region of Iraq and the rest of Iraq, to be granted the consultant level, one must practice for at least 12-13 years after receiving Board and High Diploma certification, in addition to other sophisticated processes. A plurality (n = 62/104, 59.6%) of surgeons were affiliated with the Department of Health (DoH), followed by universities (n = 35/104, 33.7%). Employment in universities has more sophisticated conditions and parameters and limited spots.

Other demographic interests include a higher number of surgeons (n = 81/104, 77.9%) working in the main cities of the Kurdistan region of Iraq than outside the cities, most likely related to better opportunities and a higher number of patients inside the main cities. Population size, job opportunities, and patient volumes lead to an increased number of surgeons in larger cities. Additionally, there are more opportunities to work in the private sector and advance your career.

Many surgeons (n = 43/104, 41.3%) managed between 101 and 500 cases (followed by n = 21/104 (20.2%) who managed between 50 and 100); these results were related to as many surgeons at the specialty level and with fewer years of experience. An abundant number of surgeons do not like performing surgery on pilonidal sinus disease due to the high recurrence rate, in addition to the low volume of cases and high competition among surgeons [17].

The most common procedure performed by our surgeons in pilonidal sinus disease is a primary open method (n = 61/104, 58.7%), followed by closed primary (n = 20/104, 19.2%). The reason is that surgeons believe that excision and the primary open method are associated with fewer recurrences and complications. However, they have a more prolonged recovery time [15,18]. Open wound healing is usually associated with a low postoperative morbidity rate, but it can be complicated by a long healing time [19]. The second most common procedure is excision and primary closure, as they believe that it has a faster recovery and less hospital stay, although there is a higher recurrence rate [15]. A study by Shakor et al. in Suleymani-Iraq concluded that the primary closure method is more comfortable for patients than the open method and is more cost-effective [20].

Minimally invasive surgeries such as laser application and phenol injections are less common among surgeons in our study. These methods are new, and many surgeons believe that they are ineffective. Reasons may also include cost and a higher recurrence rate. A study by Iesalnieks et al. states that minimally invasive procedures can be a potential treatment option for limited chronic pilonidal disease. However, the recurrence rate is higher than open healing [19].

Meanwhile, off-midline flaps such as limberg, modified limberg, and Karydakis flaps also have low frequency in this study. Although it is very effective, it is not familiar, and many consider it a sophisticated procedure that requires more practice and skills. Therefore, the low frequency of practicing these procedures in the Kurdistan region of Iraq could be primarily related to a lack of experience and skills among surgeons. Pennington et al. [18] compared closed midline with closed off-midline and revealed good evidence of higher infection and recurrence rates, slower healing, and other complications after midline primary closure than off-midline closure. A study by Iesalnieks et al. (German national guideline) states that off-midline procedures can be a primary treatment option in chronic pilonidal disease [19].

The most common procedure preferred very frequently by the surgeons in the Kurdistan region of Iraq is the primary open method, followed by the primary closure (n = 14, 13.5% and n = 10, 9.6%, respectively); this reflects that the primary open method still has very low recurrence and is a safer procedure. Reasons for performing specific procedures more frequently than other procedures are that the majority agreed on specific procedures such as excision and open method because it has a lower recurrence rate (n = 45, 43.3%), safer (n = 48, 46.2%), shorter operation time (n = 40, 38.5%), and shorter hospital stay (n = 34, 32.7%). A study by Shakor et al. showed that the treatment of pilonidal sinus disease by excision and the open method has a lower infection and recurrence rate than the closed method, but it is costly and more painful [20].

Another study by Hussein in Baquba, Iraq, concluded that the closed method is better in the sense of less pain, fewer hospital stays, and faster return to work than the open method, but has a higher recurrence rate [15]. In a study by Enshaei et al., the rotational flap is the preferred method due to fewer complications, fewer recurrence after surgery, faster healing time, and early return to work [21], which is contrary to our results, as different flap methods are very rare among surgeons in the Kurdistan region of Iraq.

Minimally invasive procedures (laser, phenol) have a very low preferable method of treatment (n = 3, 2.9%, n = 1, 1%, respectively) in this study. The reasons are that they are not safe and expensive and require experience and more training, especially the laser method. The outcome is still not established, as it is new and has limited publications. Romic et al. revealed that the use of a radially emitting laser to treat chronic pilonidal sinus disease is a novel and minimally invasive technique. Some studies, but with a small number of patients, showed promising results [22], but laser ablation for the area is one of the methods to prevent recurrence [23]. Phenol, as a minimally invasive procedure, has a limited role in the treatment of pilonidal sinus disease, as most of our surgeons do not believe in it due to its toxicity. A study by Topuz et al. in Turkey concluded that phenol treatment has not been widely used. However, it is inexpensive and better than other treatment options for pilonidal disease in terms of work-off time [24].

Regarding the point of view of recurrence, the experience of our surgeon showed that the open procedure has very low recurrence (n = 37, 35.6%). On the contrary, incision and drainage have the highest recurrence rate, but with regard to flap reconstruction and minimally invasive procedures such as laser, the majority (n =52, 50%) are not sure of its outcome. Yoldas et al., in their study, showed that the recurrence rate was 9% for flap reconstruction and 10% for the open technique [25], contrary to the experience of our surgeon.

This is the first study from Iraq and the Kurdistan region of Iraq that has studied the experience, practice, and preferences of surgeons in treating pilonidal sinus disease. However, this study has several limitations. Using an online questionnaire survey, it is not possible to select a proper random sample of the study participants, and there is also the possibility of contamination of the sample by having self-selected respondents with biases. This will affect the generalizability of the findings. Using a cross-sectional study, we cannot define causality. The use of a self-reported questionnaire leads to bias and inaccuracy in the data.

## Conclusions

At the time of writing, there is no standard treatment for pilonidal sinus disease among the surgeons in the Kurdistan region of Iraq. Most of our surgeons in the Kurdistan region of Iraq prefer the open method technique. Although our surgeons practice the easiest, safest, and least recurrent technique, it is the most painful and has the longest recovery time in treating pilonidal sinus disease. Flaps and off-midline techniques are still unfamiliar among our surgeons, although they are very effective around the world. Additionally, minimally invasive methods are very limited procedures among our surgeons, as they are new and costly and require training. We recommend that surgeons update their treatment of pilonidal sinus disease by performing more comparative research. Surgeons need more training for new methods such as flaps and minimally invasive procedures.
